# Supplementary material for: Coupled induction of prophage and virulence factors during tick transmission of the Lyme disease spirochete
Source: Nat Commun. 2023 Jan 13;14:198. doi: 10.1038/s41467-023-35897-3 (PMC9839762; doi:10.1038/s41467-023-35897-3)
Supplement: Supplementary file 1 — Supplementary Information [file 41467_2023_35897_MOESM1_ESM.pdf]

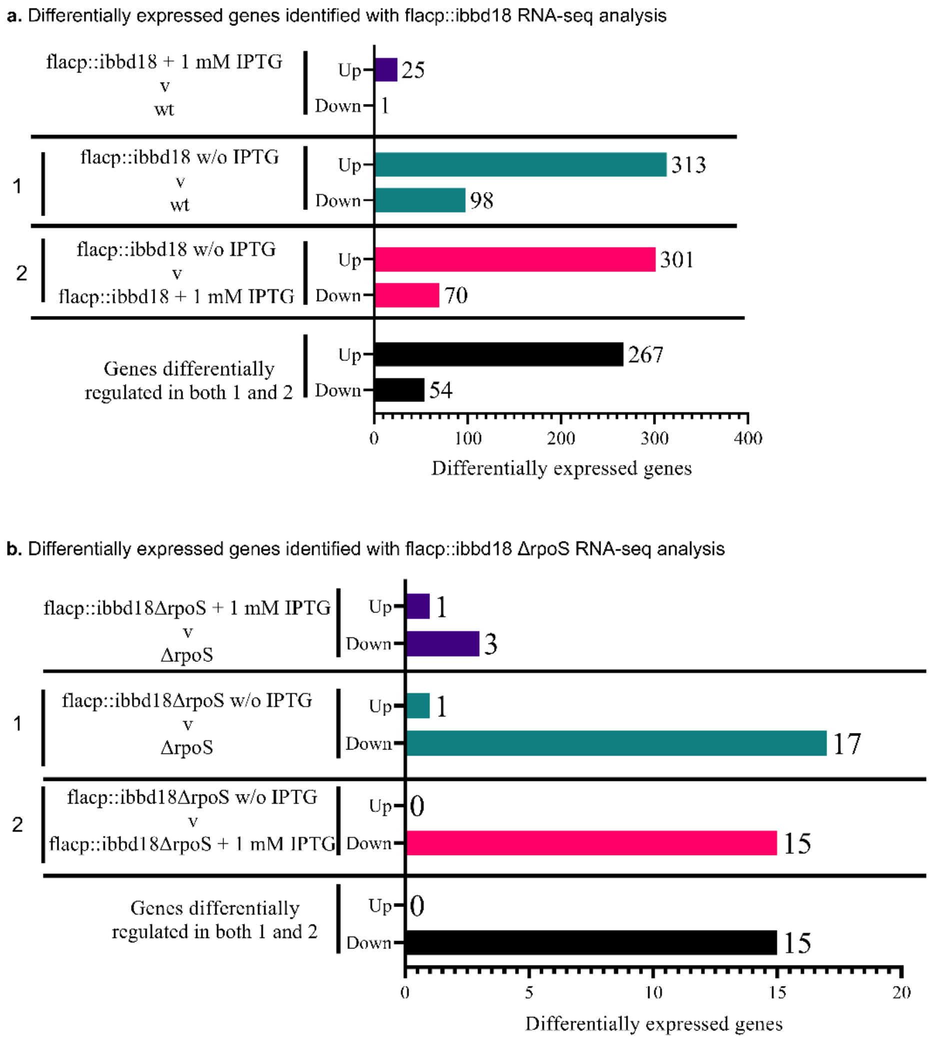

Supplemental Figure S1. Differentially expressed genes identified by RNA-seq. (**a**) The number of genes that are differentially expressed in flacp::ibbd18 cells cultured in the absence of IPTG when compared to wt or flacp::ibbd18 cells cultured in the presence of IPTG. (**b**) The number of genes that are differentially expressed when flacp::ibbd18△rpoS cells are cultured in the absence of IPTG when compared to △rpoS or flacp::ibbd18△rpoS cells with IPTG.

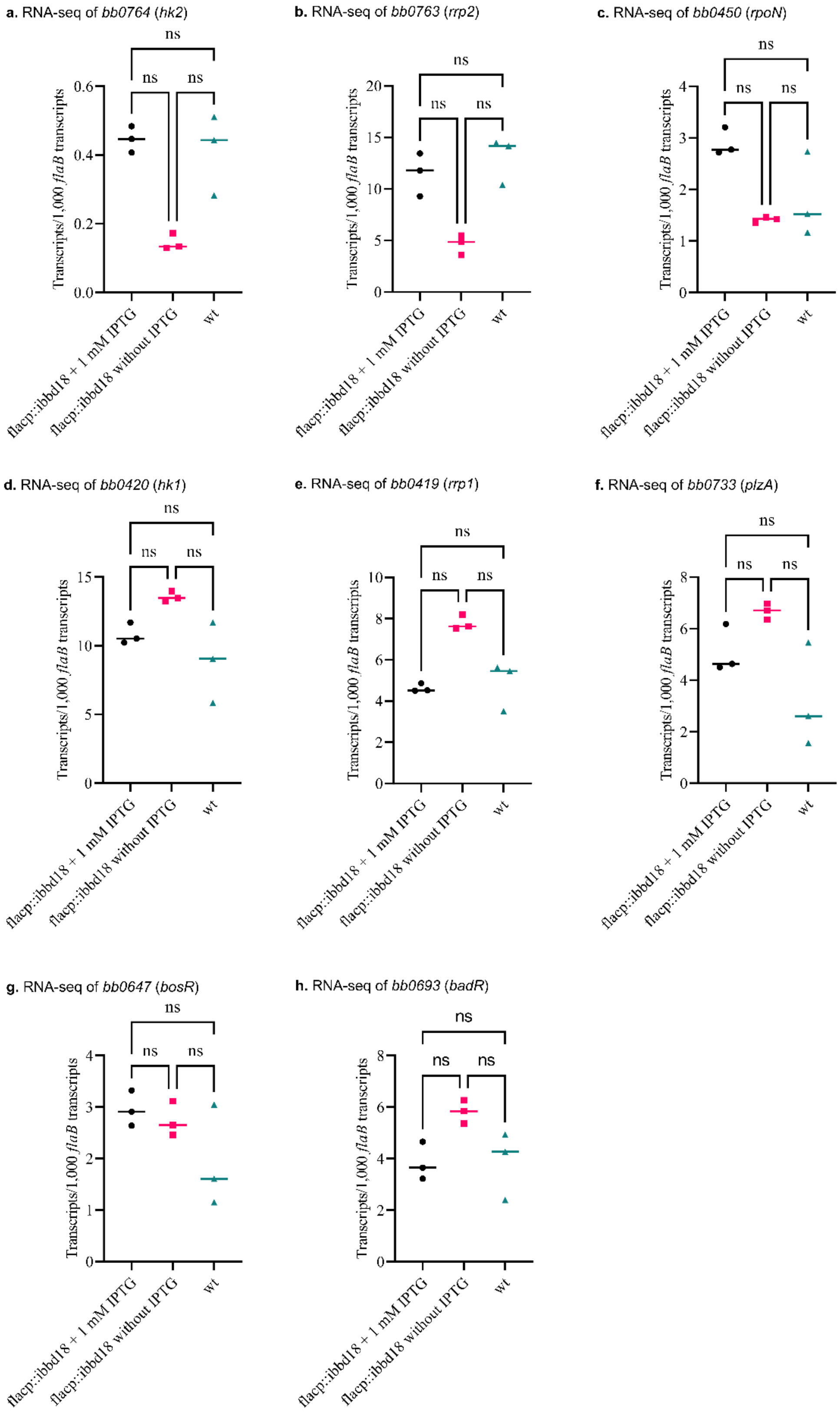

Supplemental Figure S2. RNA-seq of known *rpoS* regulators. No significant difference was found with either edgeR analysis, or when values were normalized to *flaB* and Dunn's multiple comparison of the Kruskal-Wallis test was used to determine significance. Data are presented in all graphs as individual values of biological replicates with the mean value indicated.

n = 3 biological replicates

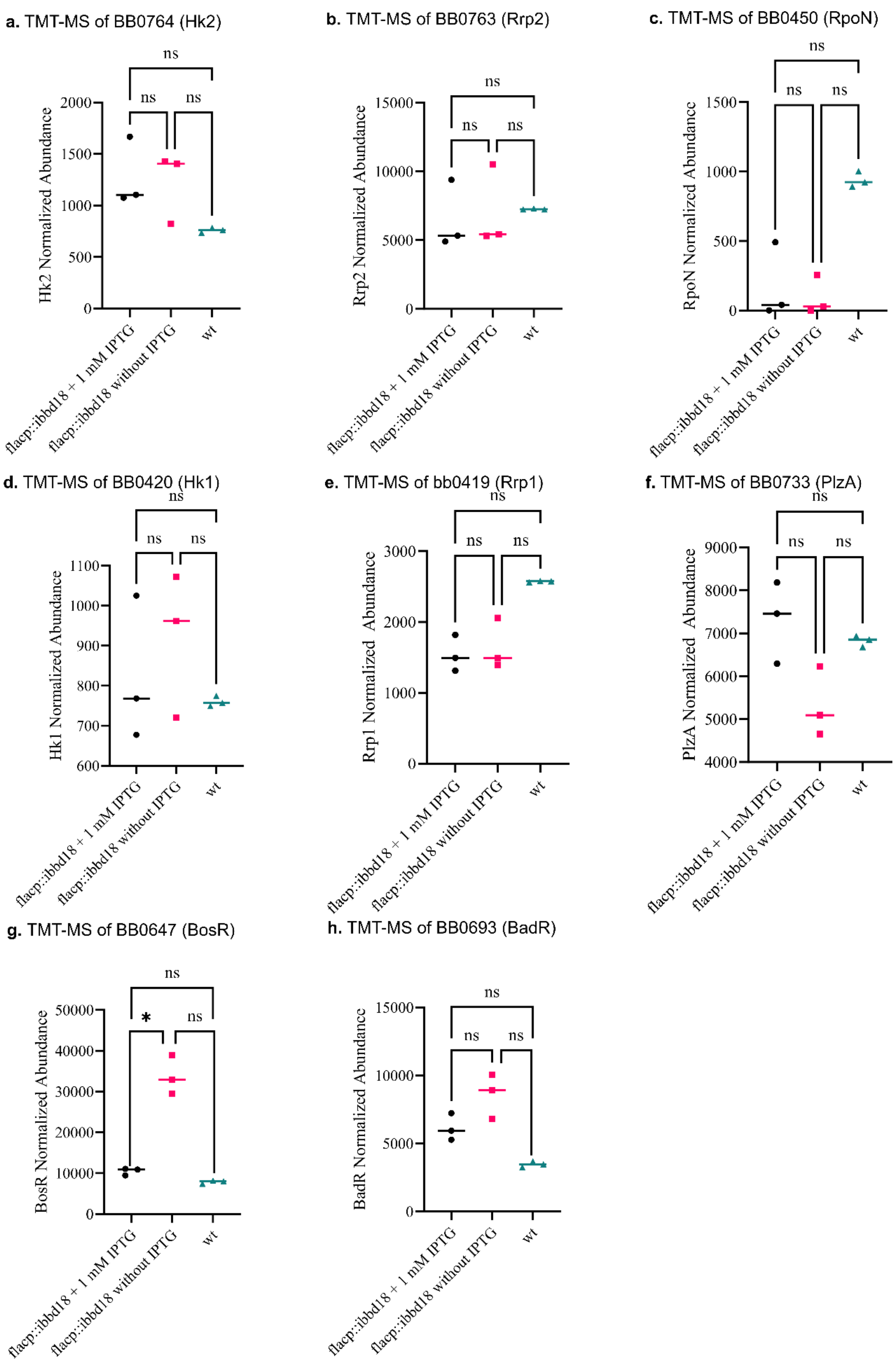

Supplemental Figure S3. TMT mass-spec of known *rpoS* regulators. Values were normalized using total intensity of all peptides approach and corrections for isotope impurities in the TMT lots were performed. Data are presented in all graphs as individual values of biological replicates with the mean value indicated. Significance was determined with a total peptide normalization and ratios calculated at the protein level.
n = 3 biological replicates, *p-value < 0.05

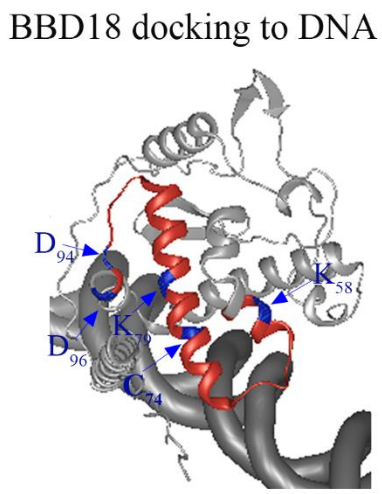

Supplemental Figure S4. Proposed structure of BBD18 bound to DNA. The BBD18 structure was modeled through AlphaFold[1,2] and DNA binding by BBD18 was added with Haddock[3,4]. Amino acid residues previously demonstrated to be important for repression of RpoS (D94, D96, C74, K79) are predicted to be at the interface with DNA as indicated in the model[5].

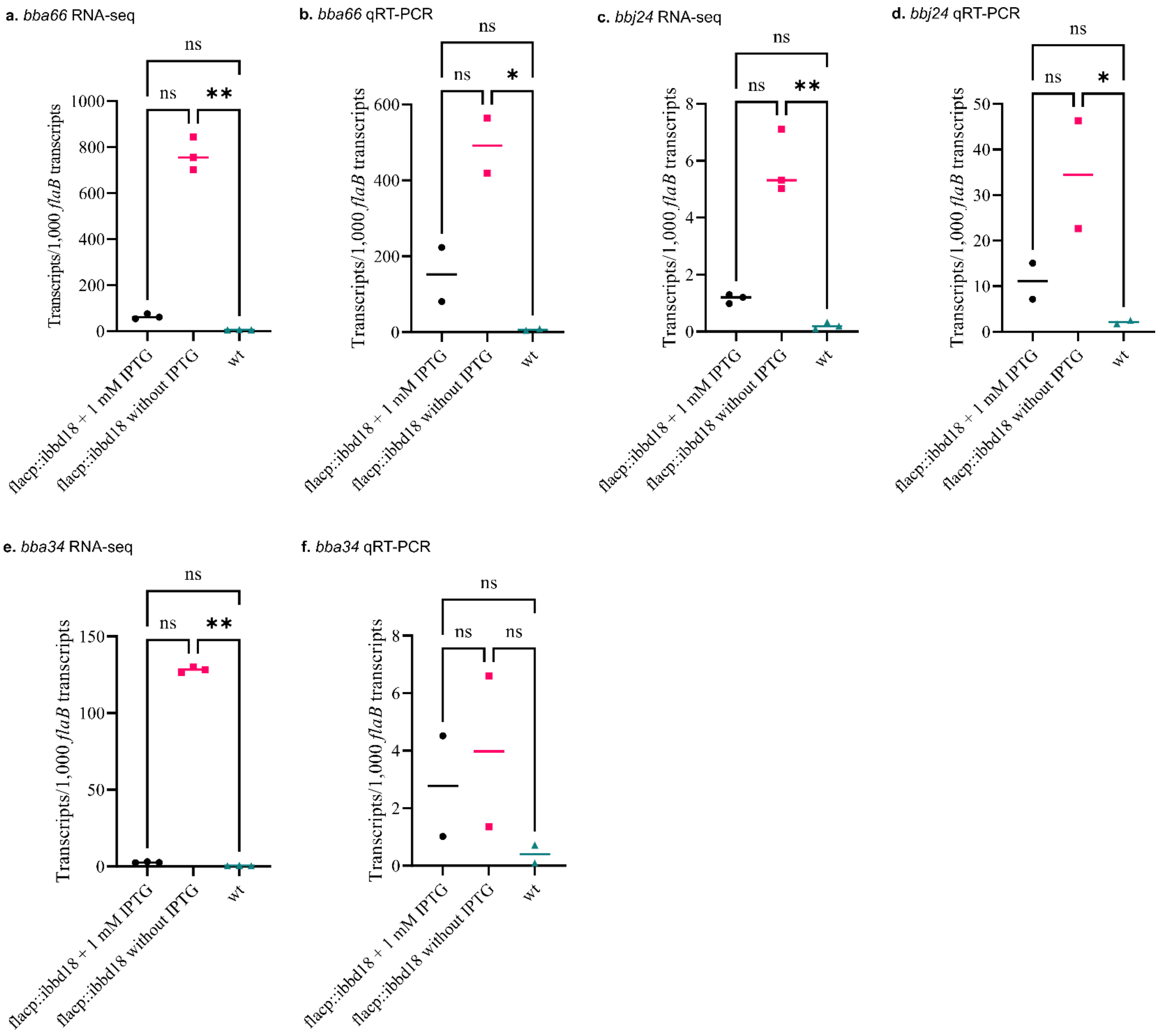

Supplemental Figure S5. RNA-seq data for a known subset of genes regulated by RpoS. Significant differences were found with both DESeq2 analysis and when values were normalized to *flaB* and Dunn's multiple comparison of the Kruskal-Wallis test was used to determine significance. Data are presented in all graphs as individual values of biological replicates with the mean value indicated.

Figures 5a, c, and e $n = 3$ biological replicates

Figures 5b, d, and f $n = 2$ biological replicates

*p-value < 0.05, **p-value < 0.005

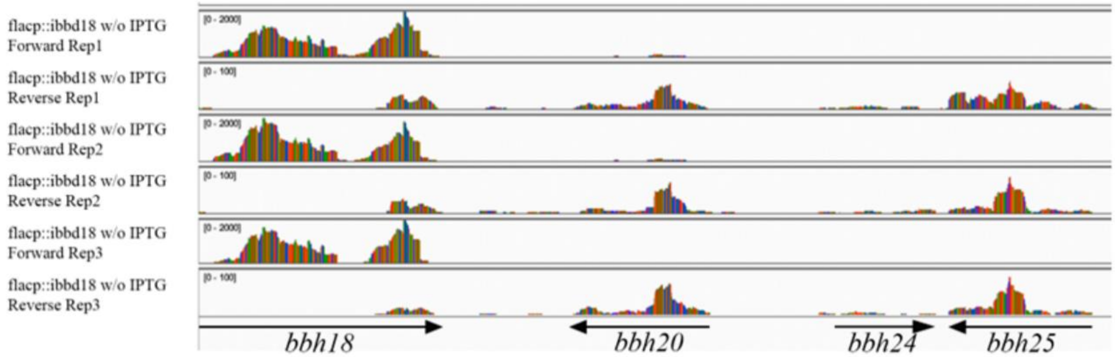

Supplemental Figure S6. Example of RNA-seq reads mapping to a segment of lp28-3. Reads mapped discretely to each gene with little to no continuation of reads between adjacent genes.

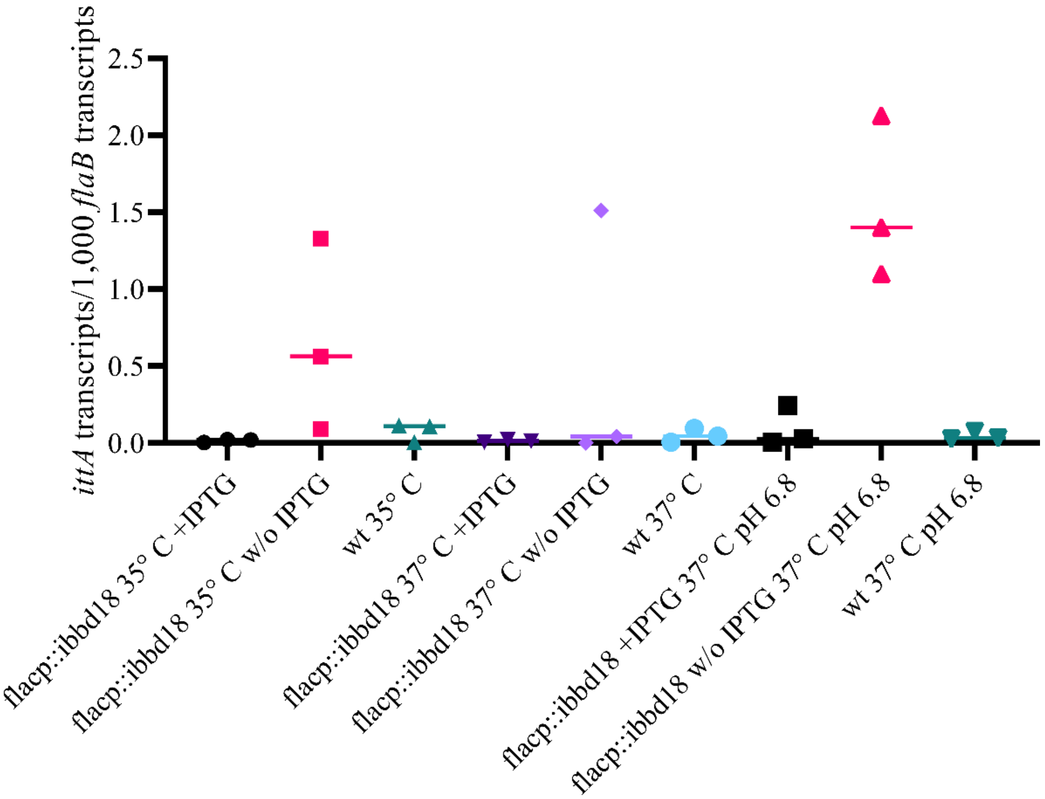

Supplemental Figure S7. qRT-PCR of *ittA* in total RNA extracted from wt (A3-68-LS) and flacp::ibbd18 cultures. *ittA* transcripts were undetectable by RNA-sequencing analysis and detected at very low levels by qRT-PCR analysis, despite the inducing conditions under which the spirochetes were cultured. Data are presented in all graphs as individual values of biological replicates with the mean value indicated.
n = 3 biological replicates

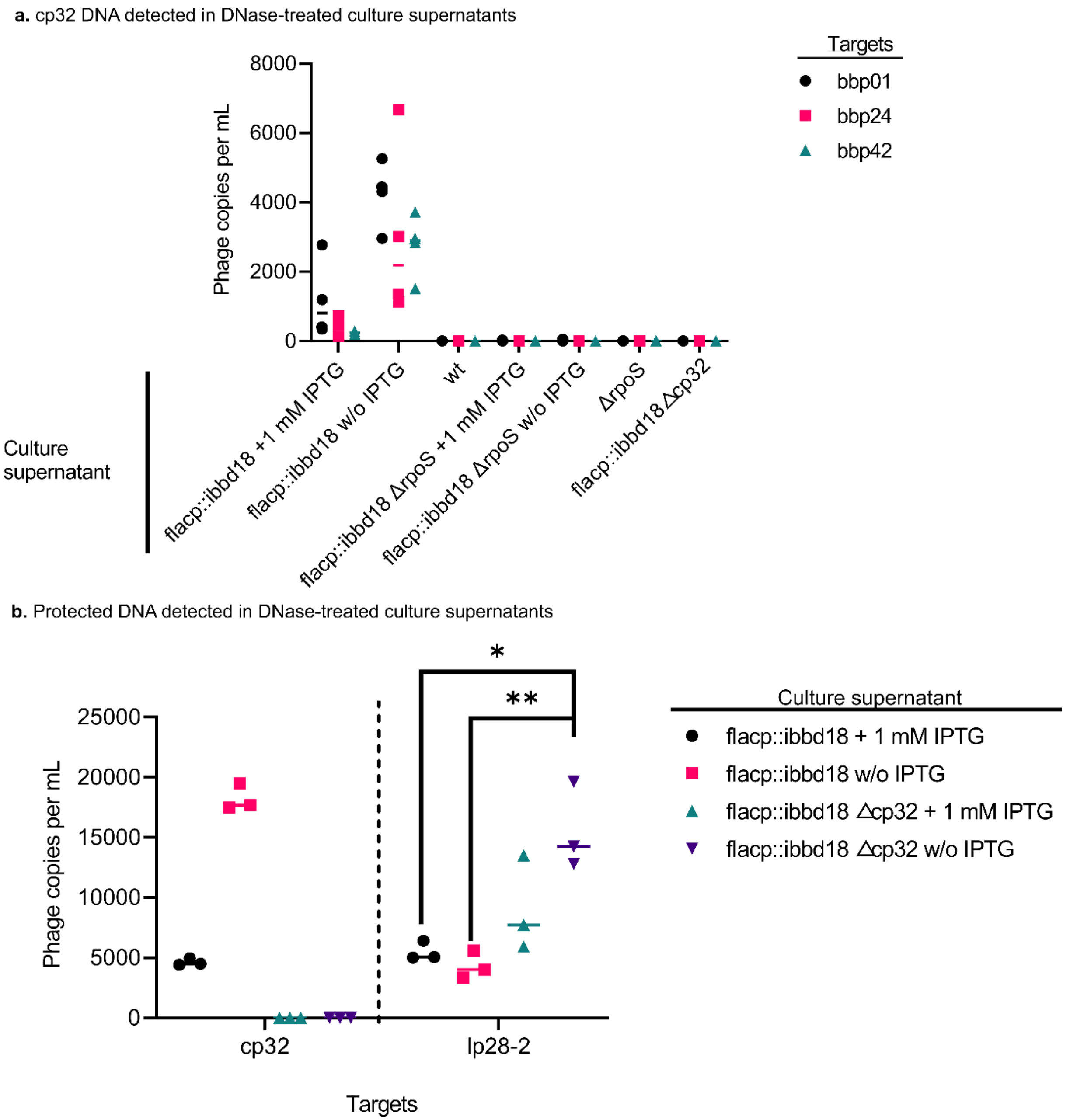

Supplemental Figure S8. qPCR of DNase-treated supernatants from wt (A3-68-LS), flacp::ibbd18, △rpoS, and △cp32 (cells lacking all cp32 plasmids) cultures. (**a**) cp32 DNA is only detected in the supernatant of flacp::ibbd18 cells. lp28-2 DNA is detected in the supernatant of flacp::ibbd18 (**b**), with significantly more lp28-2 DNA in flacp::ibbd18△cp32 supernatants. Dunn's multiple comparison of the Kruskal-Wallis test was used to determine significance. Data are presented in all graphs as individual values of biological replicates with the mean value indicated.

8a $n = 4$ biological replicates

8b $n = 3$ biological replicates

**p-value < 0.005

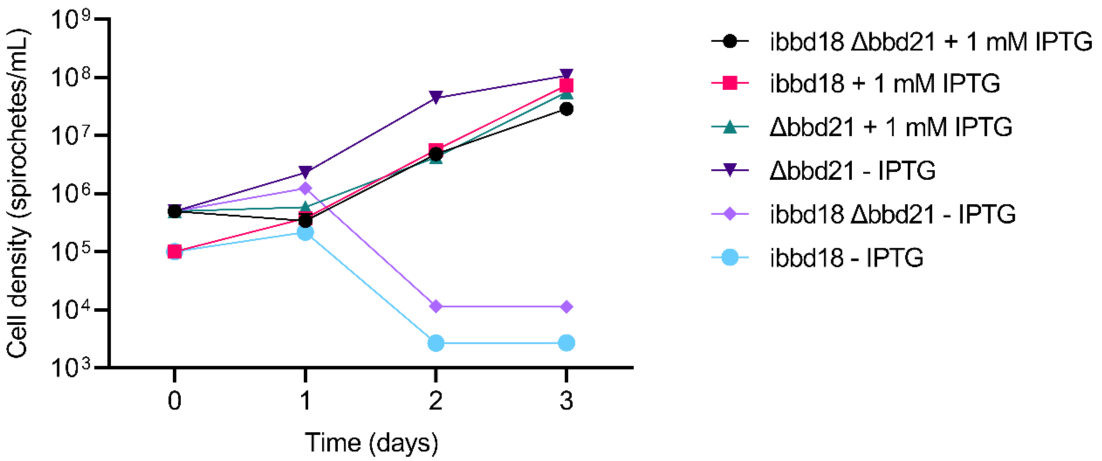

Supplemental Figure S9. Growth curve of wt, flacp::ibbd18, and △bbd21 strains. Although there is an inverse correlation between *bbd18* and *bbd21* gene expression, deletion of *bbd21* did not rescue flacp::ibbd18 cells from lysis in the absence of *ibbd18* expression (without IPTG). Data are presented as the mean values of three biological replicates +/- SD. *n* = 3 biological replicates

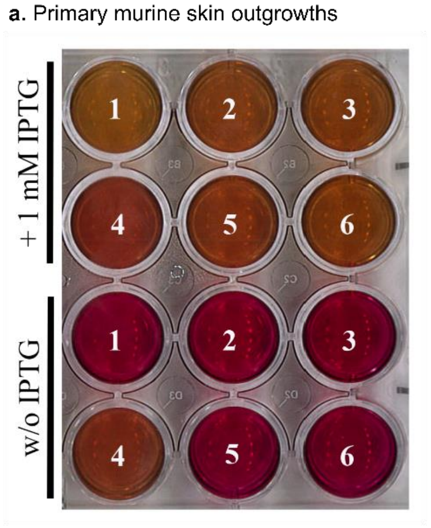

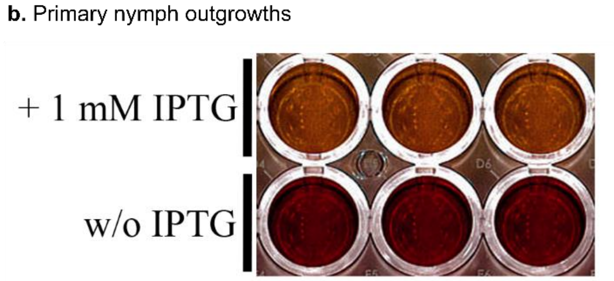

Supplemental Figure S10. BBD18 requirement for growth of flacp::ibbd18 isolates from infected mice and nymphs. (**a**) Ear punches from wt- (culture 4) and flacp::ibbd18- (cultures 1-3 and 5-6) infected mice were incubated in media supplemented with 1 mM IPTG. flacp::ibbd18 outgrowths were then transferred to media containing or lacking IPTG. Spirochete growth can be visualized by the media changing color from red (no growth) to yellow (growth). flacp::ibbd18 spirochetes isolated from infected mice require IPTG to grow *in vitro*. (**b**) flacp::ibbd18-infected nymphs were crushed and transferred to media containing 1 mM IPTG. flacp::ibbd18 outgrowths were then passed into liquid media containing or lacking IPTG. Spirochete growth can be visualized by the media changing color from red (no growth) to yellow (growth). Growth *in vitro* of all flacp::ibbd18 isolates required IPTG. This demonstrates that flacp::ibbd18 cells remain IPTG-inducible throughout infection of mice and ticks.

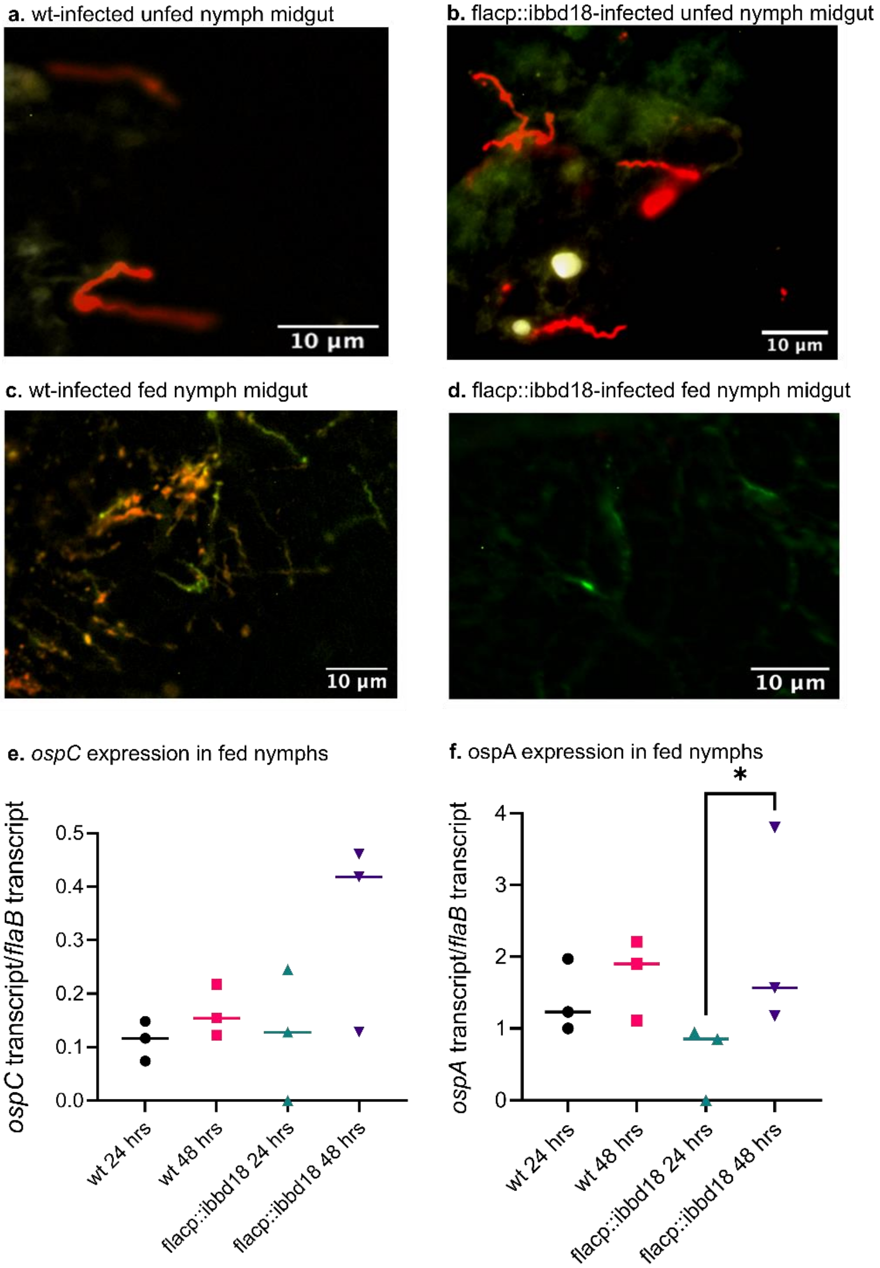

Supplemental Figure S11. Spirochetes within fed nymphs. (**a-d**) IFAs of unfed nymph midguts infected with wt (**a**) and flacp::ibbd18 (**b**) spirochetes probed with antibodies against OspC (green) and OspA (red). No OspC staining was detected in unfed nymphs infected with either strain. IFAs of midguts of fed nymphs 24 hrs after drop-off infected with wt (**c**) or flacp::ibbd18 (**d**) spirochetes probed with antibodies against OspC (green) and OspA (red). Both OspC and OspA were detected in wt-infected nymphs while only OspC (green) was detected in flacp::ibbd18-infected nymphs. qRT-PCR of fed tick midguts revealed a higher level of *ospC* transcript in flacp::ibbd18 cells compared to wt (**e**) and a similar level of *ospA* expression in flacp::ibbd18 and wt cells (**f**). Dunn's multiple comparison of the Kruskal-Wallis test was used to determine significance. Data are presented in all graphs as individual values of biological replicates with the mean value indicated.

8e-f $n = 3$ biological replicates

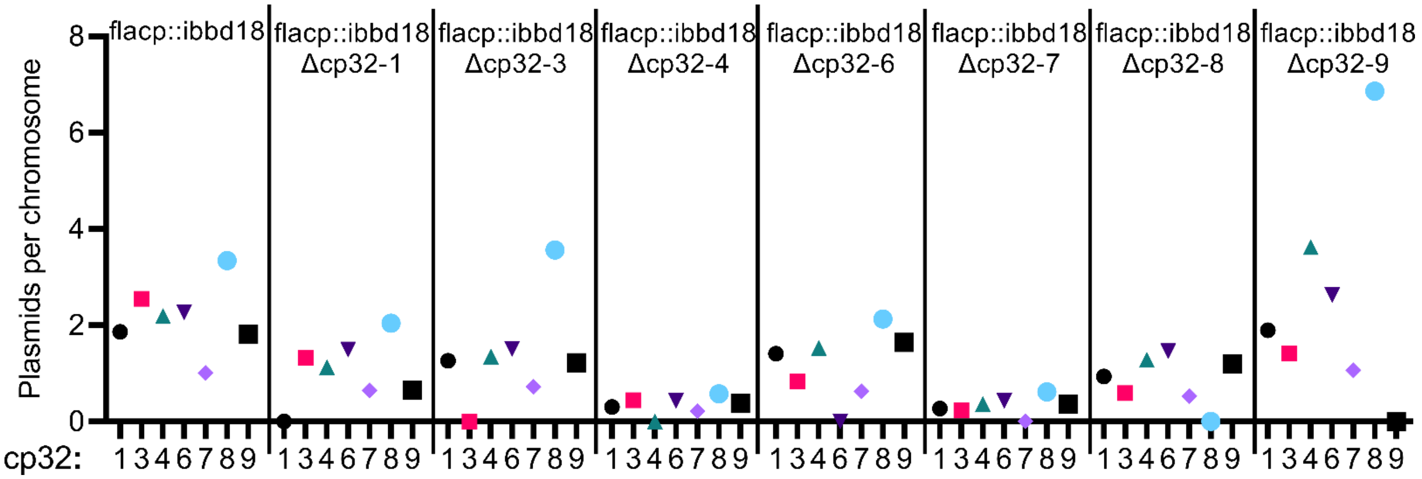

Supplemental Figure S12. qPCR of flacp::ibbd18 cells lacking cp32 plasmids. cp32 primers did not amplify a product in cells lacking the cp32 plasmid for which they were specific.
n = 1 biological replicate

<u>References</u>

1 Jumper, J. *et al.* Highly accurate protein structure prediction with AlphaFold. *Nature* **596**, 583-589, doi:https://doi.org/10.1038/s41586-021-03819-2 (2021).

2 Varadi, M. *et al.* AlphaFold Protein Structure Database: massively expanding the structural coverage of protein-sequence space with high-accuracy models. *Nucleic Acids Res.* **50**, D439-D444, doi:https://doi.org/10.1093/nar/gkab1061 (2022).

3 Dominguez, C., Boelens, R. & Bonvin, A. M. J. J. HADDOCK: a protein-protein docking approach based on biochemical and/or biophysical information. *J Am Chem Soc* **125**, 1731-1737 (2003).

4 van Zundert, G. C. P. *et al.* The HADDOCK2.2 webserver: User-friendly integrative modeling of biomolecular complexes. *J Mol Biol* **428**, 720-725 (2016).

5 Hayes, B. M. *et al.* Regulatory protein BBD18 of the Lyme disease spirochete: essential role during tick acquisition? *mBio* **5**, e01017-01014, doi:10.1128/mBio.01017-14. (2014).
