# Supplementary material for: Coupled induction of prophage and virulence factors during tick transmission of the Lyme disease spirochete
Source: Nat Commun. 2023 Jan 13;14:198. doi: 10.1038/s41467-023-35897-3 (PMC9839762; doi:10.1038/s41467-023-35897-3)
Supplement: Supplementary file 3 — Description of Additional Supplementary Files [file 41467_2023_35897_MOESM3_ESM.pdf]

Description of Additional Supplementary Files

File Name: **Supplementary Data 1**

Description: (**PacBio**) Mutations identified in the *lacI* gene and in the *B. burgdorferi* genome in wt, flacp::ibbd18 with and without IPTG and flacp::ibbd18(red) cells that underwent PacBio sequencing. (**up-regulated**) RNA-seq data of genes identified to be significantly up-regulated in flacp::ibbd18 without IPTG cells compared to either flacp::ibbd18 + 1 mM IPTG or wt cells when analyzed with edgeR. Genes previously shown to be up-regulated are shown. (**down-regulated**) RNA-seq data of genes identified to be significantly down-regulated in flacp::ibbd18 without IPTG cells compared to either flacp::ibbd18 + 1 mM IPTG or wt cells when analyzed with edgeR. Genes previously shown to be down-regulated are shown. (**TMT-MS**) Annotated proteins detected with TMT-MS and their determined abundances. (**ΔrpoS up-regulated**) RNA-seq data of genes identified to be significantly up-regulated in flacp::ibbd18ΔrpoS without IPTG cells compared to either flacp::ibbd18ΔrpoS + 1 mM IPTG or ΔrpoS cells when analyzed with edgeR. Genes previously shown to be up-regulated are shown. (**ΔrpoS down-regulated**) RNA-seq data of genes identified to be significantly down-regulated in flacp::ibbd18ΔrpoS without IPTG cells compared to either flacp::ibbd18ΔrpoS + 1 mM IPTG or ΔrpoS cells when analyzed with edgeR. Genes previously shown to be down-regulated are shown. (**animal infections**) Number of mice infected by flacp::ibbd18 or wt cells through needle inoculation or nymphal feeding and the number of fed larvae that acquired and retained flacp::ibbd18 or wt cells from infected mice. (**Strains and plasmids**) The strains of *B. burgdorferi* and *E. coli* cells used in this study and the plasmids used for the generation of constructs used in this study. (**Primers**) The primers used throughout this study. (**References**) The references for previously used strains, plasmids, primers, and data in the Supplementary Data 1 data tables.
